# Attribution of flood impacts shows strong benefits of adaptation in Europe since 1950

**DOI:** 10.1126/sciadv.adt7068

**Published:** 2025-08-15

**Authors:** Dominik Paprotny, Aloïs Tilloy, Simon Treu, Anna Buch, Michalis I. Vousdoukas, Luc Feyen, Heidi Kreibich, Bruno Merz, Katja Frieler, Matthias Mengel

**Affiliations:** ^1^ Research Department Transformation Pathways, Potsdam Institute for Climate Impact Research (PIK), Member of the Leibniz Association, Potsdam 14473, Germany.; ^2^ Institute of Marine and Environmental Sciences, University of Szczecin, Szczecin, Poland.; ^3^ European Commission Joint Research Centre (JRC), Ispra 21027, Italy.; ^4^ Institute of Geography, Heidelberg University, Heidelberg 69120, Germany.; ^5^ University of the Aegean, Department of Marine Sciences, Mytilene 81100, Greece.; ^6^ Section Hydrology, GFZ Helmholtz Centre for Geosciences, Potsdam 14473, Germany.; ^7^ Institute for Environmental Science and Geography, University of Potsdam, Potsdam 14476, Germany.

## Abstract

Flood impacts in Europe are considered to be increasing, but attribution of impacts to climatic and societal drivers of past floods has been limited to a selection of recent events. Here, we present an impact attribution study covering 1729 riverine, flash, coastal, and compound events that were responsible for an estimated 83 to 96% of flood-related impacts in Europe between 1950 and 2020. We show that, in most regions, the magnitude of flood impacts relative to the 1950 baseline has been regulated primarily by direct human actions. The population and economic value at risk have increased, but the effect of exposure growth has been largely compensated by reductions in vulnerability due to improved risk management. Observed long-term changes in climate and human alterations of river catchments were also important drivers of flood hazard in many regions, but ultimately less relevant for trends in total, continental-wide impacts.

## Teaser

Flood impacts in Europe (1950–2020) were driven mainly by changes in exposure and vulnerability, not long-term change in climate.

## INTRODUCTION

**Fig. 1.**
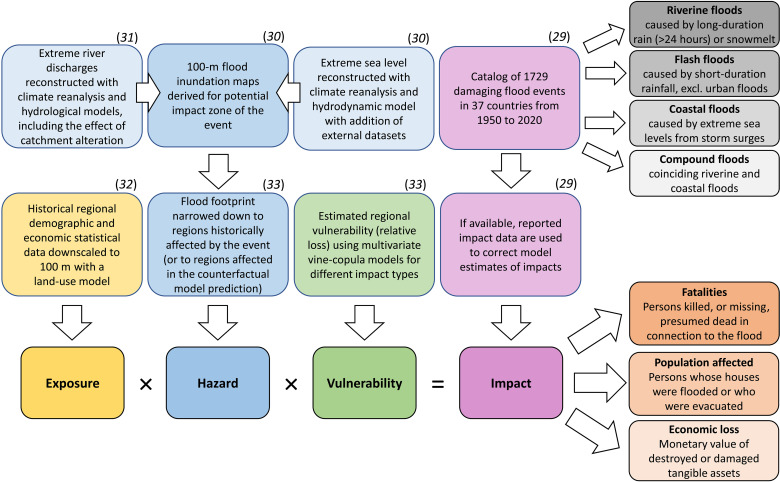
Summary of the methodology to derive impacts of historical events. The study covers three different impacts of 1729 floods, extracted from the HANZE database (*30*, *31*), belonging to four main flood types. The impacts were derived using several published models (*31*–*34*).

Floods are an ever-present risk to society and economy in Europe (*1*, *2*). Some studies attribute the increasing impacts to consequences of climate change (*3*, *4*) manifested by more extreme precipitation (*5*, *6*) and sea levels observed in recent decades (*7*–*8*). Recent decades are considered particularly rich in hydrological extremes in Europe (*2*), but the effects of long-term changes in climate on river discharges are very diverse spatially (*9*, *10*). Therefore, change in climate conditions alone cannot explain long-term variations in flood occurrence (*11*, *12*), as socioeconomic change also influences the hydrological response of catchments through reservoir construction (*13*) and land use change (*14*). Further, flood risk in Europe has been exacerbated by exposure growth (*1*, *15*–*18*) and urbanization (*11*) in riverine and coastal floodplains but reduced by flood adaptation and prevention measures (*19*–*21*). Attribution of individual European flood disasters has so far been limited to case studies, focusing on detecting the climate change component in precipitation (*22*–*24*) and rarely covering a large number of events (*25*). Only one study (*16*) estimated historic trends in all three risk components, but it used only modeled riverine flood impact time series for 1980–2010 and did not provide any information for individual countries or events. Until now, there has been a lack of consistent, detailed data on past flood events and their drivers, which are necessary for an integrated attribution of observed impacts (*11*, *26*–*28*). We address the limitations of previous assessments by combining multiple pan-European datasets to determine the contribution of six distinct drivers of impacts associated with a large number of reported flood events (*n* = 1729) from 37 European countries that have occurred between 1950 and 2020 (Fig. 1). The drivers, all evaluated against a 1950 counterfactual baseline, are (i) observed long-term changes in climate (consisting of both anthropogenic climate change and internal, natural climate variability) manifested through long-term change in river discharges and sea levels assessed primarily through nonstationary extreme value analysis (*29*); (ii) catchment alteration resulting from direct human activities—land use change, reservoir building, and evolving water demand; (iii) regional population and gross domestic product (GDP) growth; (iv) local exposure changes due to land use and evolving structure of the economy; (v) improvement or deterioration of flood prevention structures (protection levels); and (vi) change in flood vulnerability, i.e., relative loss when a flood happens, which varies due to local factors such as private precaution, building material, early warning, or emergency measures.

We have drawn reported and modeled factual (historical) impacts of 1729 riverine, coastal, and compound floods in Europe from the HANZE (Historical Analysis of Natural HaZards in Europe) impact database and its extensions (*30*–*34*). Historical flood events (*30*) were reconstructed using hydrological and hydrodynamic modeling of historical river discharges and storm surges (*31*, *32*) and historical data on land use, population, and economy (*33*) as well as a probabilistic method to estimate changes in flood protection and vulnerability (*34*). The latter includes the effect of flood experience, governance, and socioeconomic development on changes in structural protection levels and inundation impacts through adaptation. Here, we use the same models to estimate impacts of the 1729 floods under counterfactual scenarios, which represent a stationary natural and socioeconomic environment with no changes since 1950. The difference between factual and counterfactual impacts for the six drivers enables attribution of impacts (fatalities, population affected, and economic loss) to local changes in each driver. We explicitly do not attribute changes in impacts to the forced response of the climate to anthropogenic emissions but to observed long-term changes in extreme discharge, storm surge heights, wave heights, and mean sea levels induced by observed long-term changes in climate. A comparison of climate model simulations accounting for historical climate forcing versus preindustrial forcing levels has shown that observed changes in discharge are largely in agreement with the forced response (*4*) and not only driven by internal climate variability. However, our study does not try to separate these effects.

In addition, we estimate the model uncertainty to compute the likelihood that the difference between the factual and counterfactual scenario is larger than the model uncertainty. For clarity, we describe the uncertainty using the Intergovernmental Panel on Climate Change (IPCC) recommended terminology (*35*), i.e., virtually certain (>99%), very likely (>90%), likely (>66%), and about as likely as not (up to 66%). The primary unit of aggregation is 1422 subnational regions, as in Paprotny and Mengel (*33*), which, in turn, is based on European Union’s NUTS (Nomenclature of Territorial Units for Statistics) level 3 classification, v2010 (*36*)

## RESULTS

### Historical flood impacts in Europe

**Fig. 2.**
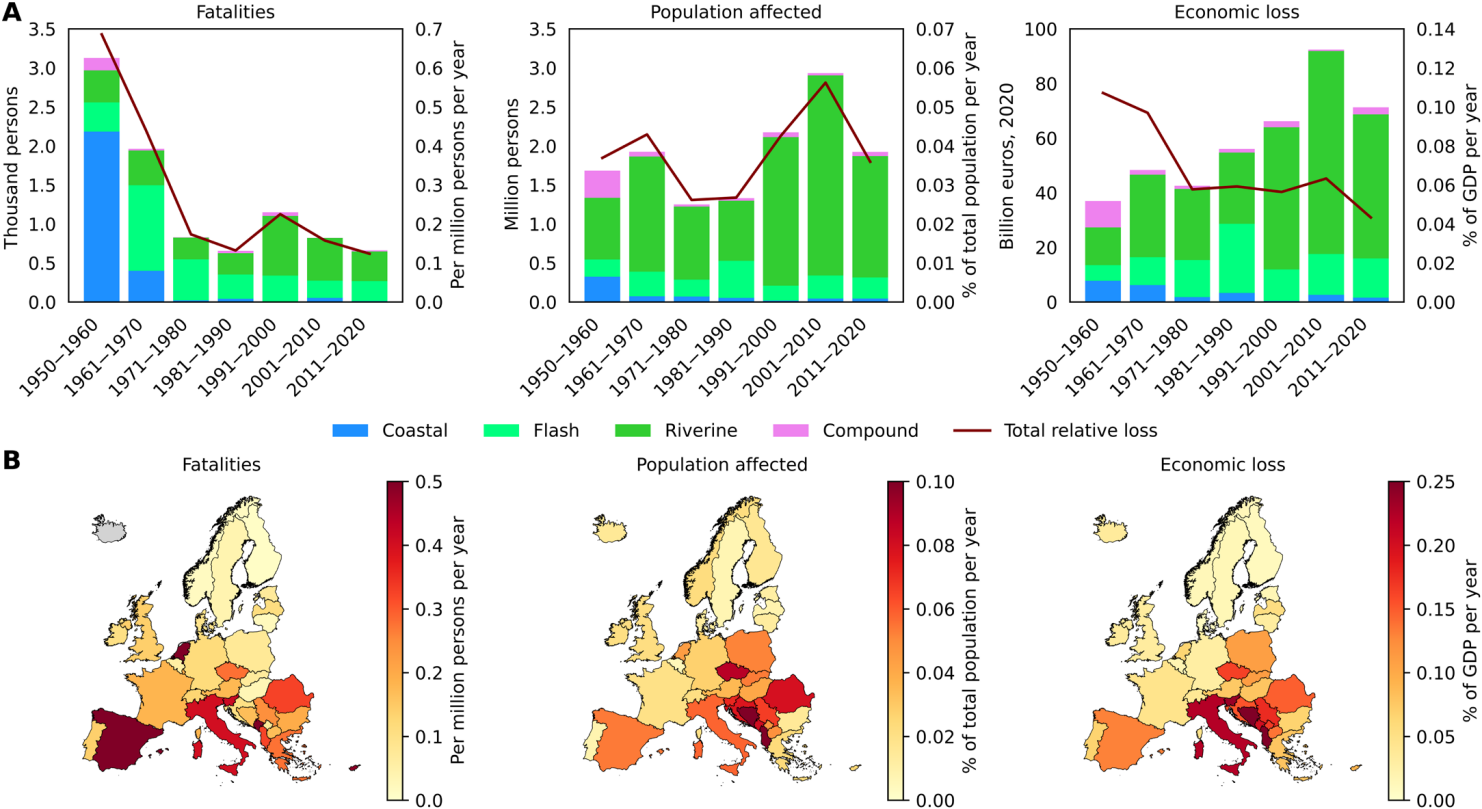
Summary of flood losses between 1950 and 2020. (**A**) Absolute flood impacts in Europe by decade and flood type, and total flood impacts by decade relative to total population or GDP. (**B**) Flood impact by country relative to total population or GDP averaged across 1950–2020. The figures include reported or estimated (if reported data were not available) impacts of 1729 floods included in this study. The number of events by type and country is shown in table S1.

Analysis of 1729 flood events shows (Fig. 2) that fatalities from flooding in Europe have declined sharply, nearly fivefold between the 1950s and the 2010s. The three main flood types (riverine, flash, and coastal) caused a similar number of fatalities, about 3000 each, but coastal flood fatalities are concentrated in the 1950s and 1960s. The number of people affected shows large temporal variability, and the upward trend is not statistically significant when evaluated at annual resolution. Slow-onset riverine floods have been responsible for the majority of people affected (76%). Direct economic losses have nearly doubled from €37 billion in the 1950s to €71 billion in the last decade. However, when expressed relative to the GDP of the study area, economic impacts have considerably decreased and are, in the last decade, about one-third of those in the 1950s. Overall impacts relative to the size of the population and economy have been most severe in southern (Spain and Italy) and southeastern parts of the continent (Albania, Bosnia and Herzegovina, and Romania), with very limited impacts in the northern parts of Europe. The majority of coastal and compound flood impacts occurred in Italy, the Netherlands, Germany, and the United Kingdom (UK), with a higher concentration in the 1950s and 1960s.

### Drivers of flood impacts in Europe

**Fig. 3.**
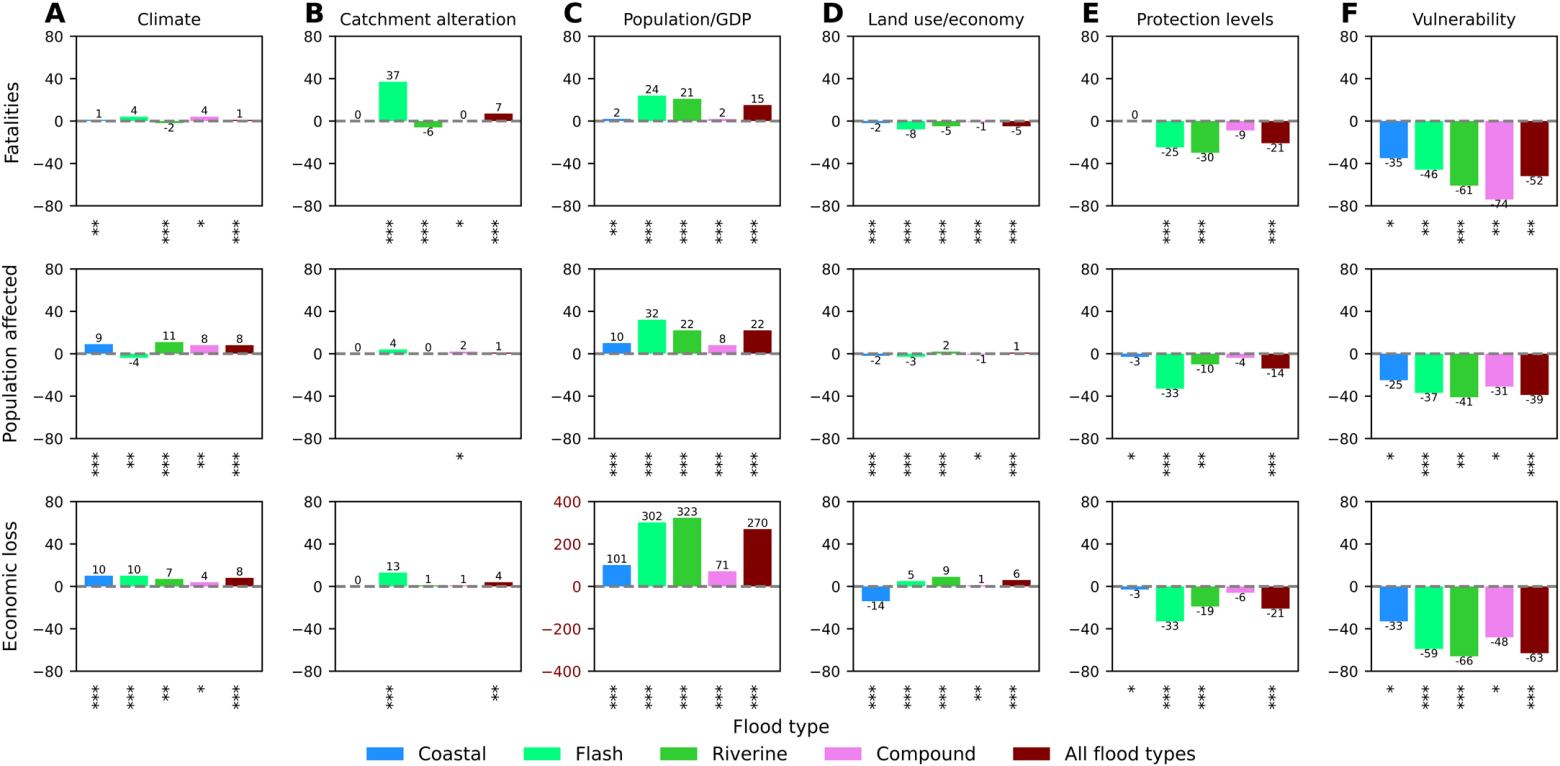
Attribution of impacts by type and driver (percent change). Contribution to impacts (in rows) of different drivers (**A** to **F**), by flood type and for all floods together, expressed as percent change of factual impacts relative to the counterfactual scenario of no change in the individual driver since 1950. Asterisks under each bar represent the likelihood that the difference between the factual and counterfactual scenario is larger than the model uncertainty: likely (*), very likely (**), and virtually certain (***).

**Fig. 4.**
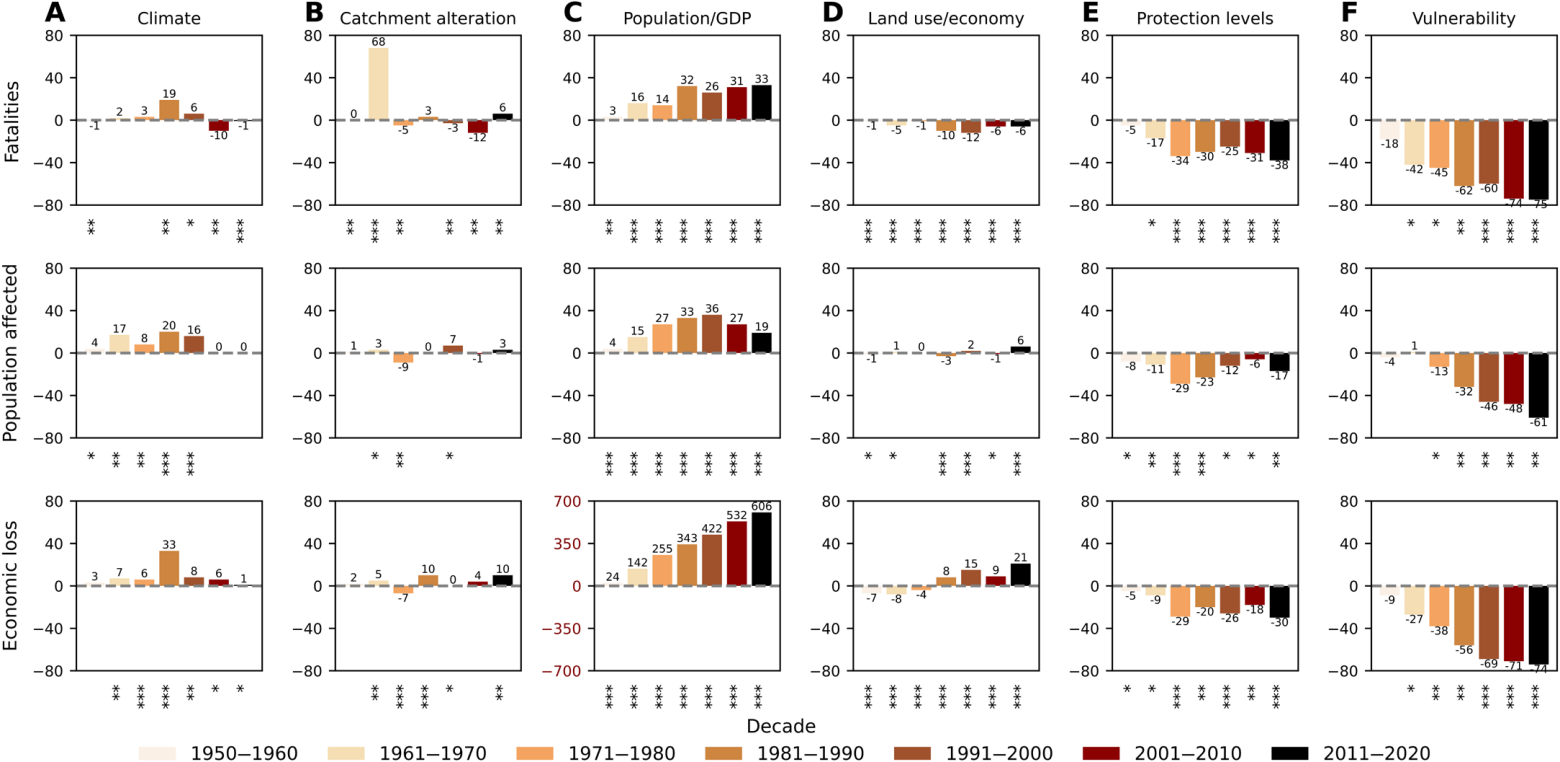
Attribution of impacts by decade and driver (percent change). Contribution to impacts (in rows) of different drivers (**A** to **F**) by decade, expressed as percent change of factual impacts relative to the counterfactual scenario of no change to the individual driver since 1950. Asterisks under each bar represent the likelihood that the difference between the factual and counterfactual scenario is larger than the model uncertainty: likely (*), very likely (**), and virtually certain (***).

The most important drivers of flood impacts in Europe have been exposure growth and vulnerability decline, with smaller contributions from other drivers (Fig. 3). It is virtually certain that observed long-term changes in climate have increased the population affected and economic loss (both by 8%) as well as increased fatalities (by 1%) (Fig. 3A). Climate has likely influenced impacts for all flood types, except fatalities from flash floods, which can be explained by high uncertainty of return period of river discharges related to those events (Fig. 2). The strongest increases due to observed long-term changes in climate were found for flash floods in terms of fatalities and economic loss and for riverine floods in terms of population affected. The impact of climate is, on aggregate, strongest in the 1980s and 1990s (Fig. 4A), before a strong decrease in extreme river discharges in the south reduces the total contribution of this factor in Europe. Catchment alteration has increased all impacts, particularly fatalities (by 7%), with population affected and economic loss increasing by 1 and 4%, respectively (Fig. 3B). For most flood and impact types, the changes due to catchment alteration are in the about as likely as not range. However, this driver is not applicable to coastal floods and the coastal component of compound floods. The most significant changes (in the virtually certain range) observed here are the increase of economic loss for flash floods by 13%, which can be attributed to strong urbanization of catchments in the past 70 years, and the decline in fatalities in riverine floods by 6%, which, in turn, can be attributed to an increase in reservoir capacity since 1950. Also, a 37% increase in fatalities from flash floods is largely due to a single high-magnitude flood in 1962 (Fig. 4B), which does not exceed the minimum hydrological threshold in the counterfactual simulation, thus indicating zero fatalities instead of 815.

Population growth at the regional level has had a much larger effect, increasing fatalities and population affected by 15 and 22%, respectively, while GDP growth has almost quadrupled economic loss (Fig. 3C). For almost all flood and impact types, the likelihood is in the virtually certain range, although the increase for coastal and compound floods is much smaller than for riverine and flash floods. This is because coastal and compound impacts have been recorded mostly in the first two decades, hence little affected by hydrological or socioeconomic trends relative to the 1950 baseline. Further, the effect on population affected has reversed after the 1990s, while, for fatalities, it has remained stable since the 1980s, as a consequence of the large slowdown of population growth in Europe (Fig. 4C).

More local exposure drivers, such as land use change and economic restructuring, had a smaller influence, although with a high likelihood (Fig. 3D). They contributed to a reduction in fatalities (by 5%) and increases in population affected and economic loss by 1% and 6%, respectively. The largest increases in population affected and economic loss were recorded for riverine floods, with more complex pictures for flash and coastal floods, while the smallest influence has been observed, for all types of impacts, for compound events. Increased economic losses due to changes in land use and the restructuring of the economy are mainly observed since the 1980s (Fig. 4D).

The last two drivers have been responsible, with high confidence, for a strong reduction in most impacts for most flood types. Flood protection, which can include a variety of structural measures, reduced impact more for flash floods (except fatalities) than for riverine floods, with the least effect on coastal and compound impacts (Fig. 3E). However, although impacts were increasingly reduced over time until the 1970s, progress seems to have stalled since the 1980s (Fig. 4E). Vulnerability reduction has been much stronger and more uniform across flood types (Fig. 3F). It is the single most important driver of change in fatalities and population affected, both overall and for each flood type. For economic losses, where the vulnerability change has been the strongest, it is only less influential than regional GDP growth (63% reduction compared to 270% increase). The reduction in vulnerability has consistently reduced flood impacts from the 1950s to the present. The strongest improvements are observed before 1990, after which they have become smaller (Fig. 4F).

### Changing flood drivers across regions

**Fig. 5.**
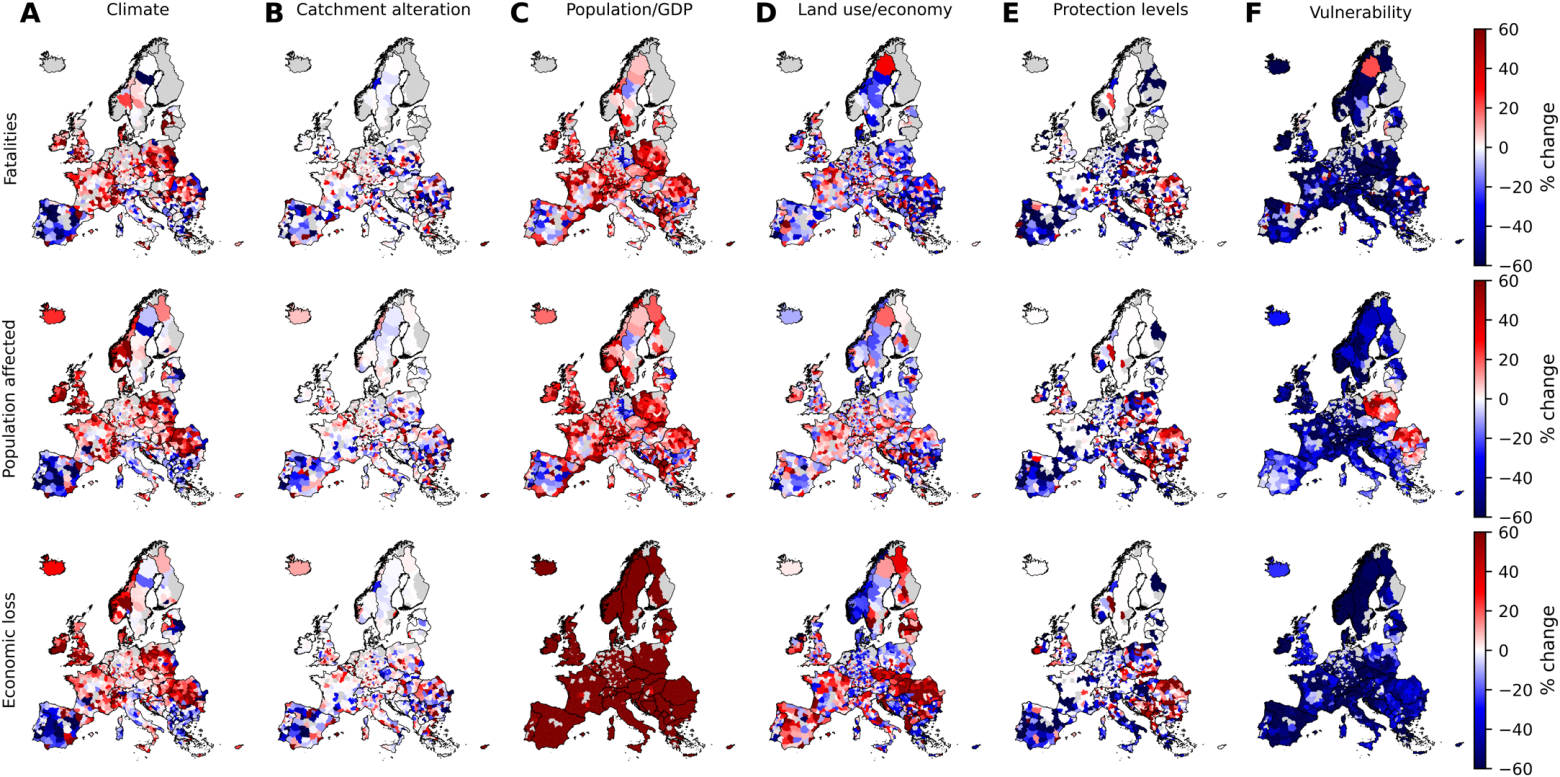
Attribution of impacts by subnational region and driver (percent change). Contribution to impacts (in rows) of different drivers (**A** to **F**), by country, expressed as percent change of factual impacts relative to the counterfactual scenario of no change in the individual driver since 1950. For aggregated data by country, see fig. S2, and for uncertainty classification by country, see fig. S3.

The different drivers of flood impacts show large spatial variation across Europe (Fig. 5). While the effect of observed long-term changes in climate on flood risk is limited when aggregated at the European scale (Figs. 3A and 4A) or even national scale (fig. S2), detailed subnational results show a clear regional divide between the north and south of the continent (Fig. 5A). In northwestern Europe (particularly the UK, Ireland, and Norway), climate has considerably increased flood impacts, while in southern Europe (particularly Bulgaria, North Macedonia, Portugal, Serbia, and Spain), the opposite effect is observed. Lower effects of climate are noticeable in northeastern Europe (Finland, Latvia, and Lithuania). In between, across western and central Europe, observed long-term changes in climate increased flood impacts to varying degrees. The effects are particularly high (with at least very likely confidence) in Greece, Portugal, Spain, and Switzerland and low (mostly likely confidence or less) in Austria, Czechia, Germany, and Sweden. No clear pattern emerges for catchment alteration (Fig. 5B), with the largest increases noticeable for Spain, Greece, and Belgium and decreases in Czechia and Serbia. In most countries, particularly in central and northern Europe, the confidence level is low (fig. S3) but is noticeably higher in many southern European countries.

Exposure growth has increased impacts in nearly all countries, with notable exceptions for fatalities and population affected in Germany and Bulgaria (Fig. 5C). However, at the subnational level, population trends can be divergent, with enormous differences, for example, between former West and East Germany or between coastal and inland regions of France and Spain. The effect of land use change and economic restructuring has no clear spatial distribution and depends strongly on the type of impact (Fig. 5D). In contrast, flood protection levels have clearly improved more in western and southern Europe than in eastern and northern parts of the continent, where some countries experience deterioration of flood protection (Fig. 5E). The highest confidence in changes across all impacts (fig. S3) is estimated in the southwest: France, Italy, Spain, and Portugal, with much less confidence in central and eastern Europe, where there is also strong variation within larger countries. Vulnerability has broadly declined across the continent (Fig. 5F), with only a few exceptions, particularly for the population affected in parts of eastern Europe (Poland, Romania, and Bulgaria).

As highlighted above, some impacts are concentrated in a small number of events, affecting primarily the attribution of fatalities. This relates mostly to the occurrence of several high-fatality events in the 1950s and 1960s, before the acceleration of climatic and socioeconomic drivers. The magnitude of the event, however, has, in most cases, a limited effect on the results when floods are classified into quintiles based on the modeled inundated area (fig. S4). A stronger influence of observed long-term changes in climate on population affected can be seen for larger-scale floods, while catchment alteration increased economic loss considerably more for smaller floods. For all types of impacts, smaller floods benefited more from flood protection. There is no clear pattern related to the size of flood for exposure or vulnerability.

## DISCUSSION

Our study disentangles the climatic and human drivers (fig. S5) of flood risk across Europe based on a large number of historical flood events spanning 71 years. Prominent among the drivers is the effect of exposure growth on increasing flood impacts as documented in the literature (*1*, *15*, *31*). Our results highlight the importance of improved flood protection and vulnerability reduction in counterbalancing the effects of rising exposure since 1950. The benefit of improved structural protection is estimated as 14 to 21% reduction of all flood impacts (and 17 to 38% in the most recent decade). However, the figure would be higher if floods fully prevented by improved defenses were considered, which is beyond the scope of this study, which only covers actual historical floods, i.e., instances where flood protection was inadequate. Still, the extent of those floods would have been much bigger and the impacts more severe than without the improvements to defenses made after 1950.

Impacts would also have been even stronger without vulnerability reduction, estimated at 39 to 63% for all floods and 61 to 75% for those between 2011 and 2020. Several factors have influenced those trends. Early warning systems were developed and improved in the last decades, enabling timely undertaking of emergency measures, including evacuation, sheltering, or deployment of temporary flood barriers (*37*–*39*). Studies have indicated better societal preparedness after major floods, related to higher awareness (through flood experience or information campaigns) and improved institutional management (through enhanced organization, planning, and civil protection units) (*38*, *40*). Individual preparedness was found to improve as well after floods through the use of various precautionary measures, e.g., building elevation, dry proofing, or wet proofing (*21*, *41*). Long-term societal and economic trends also influence vulnerability. For instance, about half of the European dwelling stock was constructed after 1950, while the share of agriculture and industry in the economy has declined from almost half to about 20%, resulting in increased share of housing and services in the fixed asset structure (*33*). As various assets have different levels of vulnerability (*42*), such structural changes alone could alter economic vulnerability over time (*34*). Population affected has experienced the lowest reduction among the impact indicators, as many vulnerability reduction measures and factors do not prevent flooding of homes but only result in lower probability of fatality and lower economic loss.

Previous studies looking at individual drivers at shorter timescales have observed some different trends than presented here. In particular, the IPCC’s latest assessment report (*7*) indicates “low confidence” that climate dynamics has influenced “change in damages” from riverine and coastal floods in Europe during 1970–2020. By contrast, we detected at least likely contributions of observed long-term changes in climate to at least some types of flood impacts for all flood types (Fig. 3A) and almost all countries (fig. S2A). We also found a spatial pattern of observed long-term changes in climate (Fig. 3A) that is broadly similar to a major study (*9*) based on hydrological observations for 1960–2010. There, the increasing annual maxima of discharges in northwestern Europe (attributed to increasing rainfall and soil moisture) are contrasted by a decline in southern and eastern Europe, attributed to decreasing rainfall and increasing evaporation in the south and decreasing and earlier snowmelt in the east. However, we find that several parts of southern and eastern Europe (e.g., Croatia, Greece, Hungary, Poland, Slovenia, and southern Italy) have experienced higher impacts as a result of observed long-term changes in climate, in contrast to downward riverine discharge trends in Blöschl *et al.* (*9*). Both limited observational data for some countries and the fact that we translate river discharge into impacts could explain the difference. For instance, discharge trends in most of Poland are driven by the reduction of snowmelt, but reported flood impacts were mostly caused by precipitation-driven summertime discharges (*30*, *43*). Sauer *et al.* (*16*), who analyzed riverine economic losses during 1980–2010, found a statistically insignificant downward trend in extreme discharge in Europe, in contrast to our finding of a virtually certain increase, albeit over a longer time period (Fig. 3A). This could be at least partially explained by a larger domain in their study, incorporating more countries with snowmelt-driven floods, which have declined due to a warmer climate, and also a much shorter time frame and a very different modeling approach. Although their study also found strong exposure growth, the vulnerability reduction was shown as much less important than in our results. Rentschler *et al.* (*18*) showed a slight increase in relative flood hazard in Europe between 1985 and 2019 due to land use change and uneven population growth, which is broadly similar to our study, but with many differences at national and regional level (Fig. 4D). Mazzoleni *et al.* (*11*) also highlighted the strong influence of both climate change and catchment alteration on floods along major European rivers but without the spatial variation observed in our study.

In this study, we only assessed observed trends in riverine and coastal water levels due to long-term changes in climate and not specifically to anthropogenic climate change, therefore including internal climate variation (*44*, *45*). This results from our approach to use climate reanalysis (*46*) and nonstationary extreme value analysis (*29*), which enables detection of climate trends and their influence on specific historical events but does not determine the influence of external forcing on those changes. Still, a comparison of climate model simulations accounting for historical climate forcing versus preindustrial forcing levels has shown that observed changes in discharge are largely in agreement with the forced response (*4*) and not only driven by internal climate variability. The patterns of high river discharge shown for Europe in that study are similar to those presented here, namely, a decrease in the south and an increase in the central and northern parts, but in much higher resolution.

Relatively few studies have quantified the contribution of observed long-term changes in climate to observed impacts at flood-event level using hydrological models and/or risk analysis (*27*) but nonetheless could be compared with our results. Pall *et al.* (*47*) found that the risk of the autumn 2000 floods in the UK very likely increased by more than 20% due to climate change and likely much more. Our findings show a very likely contribution of observed long-term changes in climate to impacts of that event, 32 to 33% above the counterfactual, depending on the type of impact considered. Schaller *et al.* (*48*) found a 21% increase in risk of extreme discharges in the Thames catchment (UK) for the 2013/2014 floods, translating into about 1000 additional properties affected. Kay *et al.* (*49*) refined the estimate to 457 additional properties flooded due to climate change, although the baseline number of properties is not mentioned. We estimate that the riverine component of the 2013/2014 floods across the UK affected 18% more people than in the counterfactual that removes the observed long-term changes in climate. However, the uncertainty is high, which was also found in the aforementioned case studies.

Last, our results on the multifactor attribution of floods during 1950–2020 put into context more recent floods, the attribution of which has been limited to meteorological drivers only (*24*, *25*, *50*). In 2021, western and central Europe was heavily affected (*51*–*53*), likely making it the costliest year as a share of GDP since 1966 and the most fatal since 1973. On the other hand, 2022 has been one of the least impactful years since 1950, while 2023, due to major impacts in Greece, Italy, and Slovenia (*51*, *54*), was again above average in terms of economic loss relative to GDP, but not highly unusual. The year 2024 brought further flooding in central Europe (costliest since 1997) and Spain (deadliest since 1973), leading to European impacts not far from those in 2021 (*51*, *55*). This is because exposure growth will have contributed strongly to the absolute value of losses, creating the impression of exceptional magnitude of impacts, which is less important in relative terms. Still, the observed vulnerability reduction over past decades also shows that the impacts of those events would likely have been much higher if they had occurred earlier. In the future, sea level rise and extreme precipitation are expected to increase in most parts of Europe (*4*), and exposure growth will continue to increase losses as well (*56*, *57*). More efforts to improve flood protection, reduce vulnerability, and limit exposure growth are therefore needed. This can be achieved through a combination of structural measures, private precaution, relocation, and nature-based solutions aimed at improved retention (*58*). In this study, we did not cover possible long-term, nonlinear interactions between hazard, exposure, flood protection, and vulnerability. Phenomena such as “levee effect” (elevated exposure growth due to perception of safety behind flood defenses) or “adaptation effect” (uptake of vulnerability-reducing measures after flood impacts) are well described (*59*, *60*), but including such effects in attribution will require advanced sociohydrological modeling (*61*).

## MATERIALS AND METHODS

### Records of historical floods

The dataset of 1729 historical floods analyzed here is a model reconstruction of past floods (Fig. 1) (*31*–*34*), but every modeled event represents a flood known from documentary sources to have occurred historically and causing socioeconomic impacts. Two sources identify those historical floods and provide their date, location (by subnational regions), type, and, if available, reported impacts (fatalities, population affected, and economic loss). Most floods were identified using the HANZE database, v2.1 (*30*). That dataset was constructed with more than 800 sources of information ranging from news media through government reports to scientific papers, providing information on 2521 floods that caused socioeconomic impacts between 1870 and 2020. Of these, 2037 events occurred within this study’s time frame (1950–2020), which is limited by the availability of climate reanalysis data [ERA5-Land (*46*) starting in 1950]. With the use of hydrological and damage modeling (described below), 1504 events were reconstructed by Paprotny *et al.* (*31*). A reported event was considered matched with modeled floods if both hydrological and impact thresholds defined in the study (*31*) were reached in at least one NUTS3 region that was affected by a reported event within the same time frame as that event. Not all events had all the impact data available (i.e., fatalities, population affected, and economic loss), but every event was described by at least one type of impact. In such situations, impacts were gap-filled with model estimates made in Paprotny *et al.* (*34*), also described below. In addition to the HANZE database, 237 floods without impact data were identified in Paprotny *et al.* (*31*). Those floods were considered damaging above the impact thresholds set in HANZE based on qualitative or partially quantitative data from documentary sources. We exclude here 12 events that represent a phase of a flood event from the HANZE database, which was indicated in the model as a separate event, but whose impacts were already included as part of the “main” model event connected with HANZE. The remaining 225 were included in our study, resulting in a total of 1729 flood events available for analysis. Paprotny *et al.* (*34*) estimated that those 1729 events cover approximately 83% of fatalities, 96% of the population affected, and 95% of direct economic losses from flooding in Europe between 1950 and 2020 (table S2). Because of the resolution of meteorological data and hydrological models, it was not possible to include, in particular, many small or rapid flash floods.

#### General approach

Each historical event in the study was reconstructed using a set of connected, pan-European models of hazard, exposure, and vulnerability (Fig. 1). The factual impact estimate of each event, i.e., representing conditions of each driver at the time of event, was obtained from previous research (*30*–*34*). We use the same models as were used to derive the factual estimate to recreate each flood under counterfactual scenarios representing the climatic and socioeconomic conditions of the year 1950. We present here six of 64 possible scenarios, in which only the driver in question is set to be counterfactual, while all other drivers remain factual. Uncertainty related to a given driver is analyzed on the basis of the confidence interval of the model related to that driver. For each event, there are one or more subnational regions affected. Factual impact in each region is either the reported impact disaggregated proportionally to modeled impacts or directly the prediction from the models. Where disaggregated reported data were available, counterfactual impact per region is corrected proportionally to the ratio between disaggregated reported impact and the modeled impact. Total impacts per event are computed by summing regional-level impacts.

The methodology of deriving each driver is summarized in fig. S6. Below, we describe the methodology separately for each driver, noting the different approaches related to observed long-term changes in climate depending on the type of floods.

### Observed long-term changes in climate (riverine and flash floods)

Riverine flood events were derived from Paprotny *et al.* (*31*), which were, in turn, an extension of the Hydrological European ReAnalysis (HERA) dataset (*32*). HERA provided river discharge for Europe simulated for the period 1950–2020 using the state-of-the-art hydrological model LISFLOOD (*62*), with spatial resolution of 1 arc min (1.8 km at the equator) and temporal resolution of 6 hours. The model was forced with climate reanalysis data [ERA5-Land (*46*)], bias-corrected and downscaled to the model resolution with weather observations using the ISIMIP3BASD method (*63*). Grid cell–level extreme river discharges along river segments with an upstream catchment area of at least 100 km^2^ were aggregated to create spatially and temporarily coherent flood events (*31*). Then, maximum river discharge at each grid cell was converted into an inundation zone within the nearest river segment. Flood inundation maps were obtained from previous hydrodynamic simulations (*64*, *65*) for different scenarios corresponding to a defined discharge amount. It was therefore possible to find the inundation maps corresponding to river discharge from HERA, with interpolation between flood maps created for higher and lower discharges where necessary. Only regions with sufficient potentially flooded area and events with sufficient impact potential [based on population and asset value maps (*33*)] were included in the final dataset of 11,205 riverine floods (*31*), of which 1592 corresponded to impactful historical floods and were included in this study.

Counterfactual flood event modeling repeats the abovementioned approach but using detrended river discharges. First, river discharge was simulated with the LISFLOOD model considering factual climate but static 1950 socioeconomic conditions (see the “Catchment alteration” section below), resulting in discharge time series that represent only the effects of long-term changes in climate. Extreme discharges were then detrended using the transformed-stationary extreme value analysis (tsEVA) approach (*29*). The ratio between discharge with and without detrending was then used to correct grid cell–level discharge in the factual HERA simulation (*32*). Last, the river discharge under the counterfactual climate scenario was aggregated into flood events and converted to inundation zones with the exact approach used to create the factual event dataset (*31*). This approach assumes that long-term changes in climate had the same proportional effect in a given grid cell both at the 1950 level of catchment alteration and at historical levels of that alteration, therefore ignoring some possible nonlinear catchment responses to long-term changes in climate.

The applied tsEVA method decouples the detection of nonstationary patterns from the fitting of the extreme value distribution with a generalized Pareto distribution (GPD). It was applied at every riverine grid cell as described by Mentaschi *et al.* (*29*), only adjusting the parameters of the transformation. First, a time series of extreme riverine flows is created, using discharges above the 95th percentile (in 6-hourly resolution) and a minimum distance between flood peaks of 3 days (actual distance is determined by continuous exceedance of discharge threshold). Then, using a moving 30-year time window (typical time frame for climate analyses), the long-term changes in mean and standard deviation over time were detected. In addition, the seasonal (monthly) cycle of discharges is detected and used to vary the mean and standard deviation changes by month of the year. This enables transforming a nonstationary time series into a stationary one, on which the GPD distribution can be applied. After the extreme value analysis, the result is reverse transformed into a nonstationary GPD distribution. The scale and location parameters of the resulting distribution are time varying (both long-term and seasonally), but it is still assumed that the shape parameter does not change over time. This assumption does not consider the possibility of time-varying skewness or kurtosis of the time series. However, this would increase the degrees of freedom, thus further increasing the uncertainty of the analysis. The method was used to convert extreme discharges at a given return period, computed with the tsEVA approach at every riverine grid cell and assessed at the time of the event under historical climate, to the discharge for the same return period in 1950.

To compute the likelihood that the modeled impacts under the factual scenario are different from the counterfactuals, we quantified the uncertainty in estimating nonstationary parameters of the GPD distribution. The analysis is simplified as follows to make it computationally feasible. The standard error in trends of average and standard deviation of extreme river discharges is the basis of standard error in the GDP parameters in the tsEVA approach (*29*). For every extreme discharge event, the relative difference in discharge due to error of fit was calculated and an average difference was computed for each NUTS3 region. We assume that the uncertainty in extreme river discharge can be represented by a normal distribution with a location parameter of zero. The scale parameter of the distribution is assumed to be equal to the mean absolute error in discharge due to uncertainty of the nonstationary GPD fitting. Then, a transformation factor of discharge uncertainty into impact uncertainty was estimated per NUTS3 region using available flood hazard maps and exposure maps for 2020. Impact magnitude at different discharge values (corresponding to different return periods of hazard maps) was calculated with the vulnerability model (*34*). The results were converted into a linear function between extreme discharge deviation and impact magnitude change. In this way, the uncertainty distribution of discharge could be sampled and then converted into a percent deviation of flood impacts from the mean prediction of impacts made by combining hazard, exposure, and vulnerability models under any scenario (Fig. 1).

### Observed long-term changes in climate (coastal and compound floods)

Similarly to river discharge, the coastal flood dataset was constructed by combining a set of extreme sea level events with coastal flood maps, as described by Paprotny *et al.* (*31*). To obtain sea levels, we carried out a Delft3D-based simulation of storm surges along European coasts (*66*), driven by ERA5 reanalysis data (*67*). Hourly storm surge heights were combined with data on tidal elevations from the FES2014 model (*68*), significant wave height (converted to wave run-up) from ERA5, long-term sea level rise, mean dynamic topography of the ocean, and glacial isostatic adjustment from the ICE-6G_C model (*69*, *70*) to estimate the total water level. Long-term sea level rise after 1950 is represented by annual sea level data for 1950–1999 from a high-resolution reconstruction (*71*) and for 2000–2020 from satellite altimetry (*72*, *73*). On the basis of those water levels, flood hazard maps were computed in Paprotny *et al.* (*31*) according to the methodology of Vousdoukas *et al.* (*74*) to derive footprints and water depths and were combined with sea levels as in the riverine flood events. This resulted in 2436 potential coastal floods, of which 88 corresponded to historical damaging floods and are analyzed here. In addition, where potential riverine and coastal floods co-occurred in the same NUTS3 region, a separate “compound” event was created (1058 events in total, of which 49 are analyzed here). Compound flood events are a combination of riverine and coastal events; therefore, factual and counterfactual drivers for the two components were first computed according to their respective methodologies, and the resulting inundation zones were merged by taking the maximum water depth out of the two layers.

Counterfactual coastal floods were computed by modifying three components of extreme sea levels and then recalculating inundation zones and impacts. Hourly storm surge height and significant wave height were calculated for 1950 using the tsEVA approach, similarly to river discharges, as described above. However, parameters of the transformation were modified using 99th percentile (due to higher temporal resolution and shorter duration of coastal events) and not using seasonal changes (as it is less relevant here and allows more confident estimation of return periods). In addition, long-term sea level rise after 1950 added to factual sea level was subtracted in the counterfactual. Uncertainty was computed in the same way as for riverine floods, i.e., uncertainty of factual and counterfactual extreme sea level is assumed to be the result of the uncertainty of the nonstationary GPD fitting.

### Catchment alteration

The factual river discharge simulations with LISFLOOD used variable socioeconomic input maps for every year of the simulation (*32*). This accounted for new reservoirs (based on the year of construction), land use changes (six classes defined in LISFLOOD: rice, other irrigated land, forest, sealed surfaces, open water, and other, i.e., nonirrigated agriculture, nonforest natural, and pervious artificial), and water demand changes. The river discharge simulation was repeated with fixed socioeconomic input maps (land use, reservoirs, and water demand) for the year 1950 to derive counterfactual discharge. In case of water demand, the intra-annual (monthly) cycle for the domestic and energy sectors was preserved as it varies depending on the temperature distribution within the year. Hence, the seasonal cycle of each individual year is maintained. The time series was scaled proportionally so that the annual totals of water demand per sector were reduced to the 1950 level. Uncertainty calculation followed the same methodology as for observed long-term changes in climate.

### Population and economic growth

This driver represents the changing population and GDP at the level of subnational regions. We use a harmonized dataset of historical socioeconomic statistics, based on almost 400 different data sources created by Paprotny and Mengel (*33*) to derive the factual value of regional-level exposure. The counterfactual scenario fixes population and GDP at 1950 levels. We assume that the uncertainty of population and GDP can be represented by a normal distribution with a location parameter of zero. The scale parameter of the distribution was assumed to be 2% for population. In the case of GDP, the parameter amounted to 10% in 1950 and declined by 0.1 percentage point per year, reaching 3% in 2020. The value for population is based on scarce postenumeration evaluations of population census in developed countries (*75*–*77*), which indicate that the error of those is usually less than 2%. The 3% value for GDP in 2020 is equivalent to the average revision of historical GDP figures in European countries when transitioning from the European System of Accounts (ESA) 1995 to the ESA 2010 system of national accounts [based on current and archived Eurostat data (*33*)]. The 10% figure is roughly equivalent to the revision of GDP figures for European countries in 1950 between the 2010 and 2023 iterations of the Maddison Project Database of historical GDP estimates (*78*). It also reflects the lower availability of subnational GDP data in early decades compared to almost complete data in the recent two decades.

### Land use and economic structure

This driver combines spatial and structural changes in exposure. That includes land use change (affecting spatial distribution of population and assets), change in the value of fixed asset stock relative to GDP, and change in the structure of fixed asset stock due to the evolving structure of the economy. High-resolution (100-m) historical land use, population, GDP, and fixed asset values were also obtained from Paprotny and Mengel (*33*). The modeling approach combines high-resolution predictor maps for changes in exposure distribution with rule-based and statistical methods to downscale regional-level exposure. The dataset can capture historical changes in the spatial redistribution of exposure due to population and economic growth, land use change (especially through urbanization and building of new infrastructure), change in the structure of the economy (GDP and asset composition), and change in asset-to-GDP ratio per economic sector. In the counterfactual scenario, 100-m maps of population and fixed asset value by sector for 1950 are used. The uncertainty was sampled from the uncertainty bounds (nearly uniformly distributed) generated by the exposure model by Paprotny and Mengel (*33*) when applied to the flood footprint of each event. The model includes the uncertainty of changes in population distribution due to urbanization, modeled with copulas [as described in (*33*)], and the uncertainty of land-use transitions, modeled with a Bayesian network [as described in (*33*)].

### Flood protection levels

In the factual scenario, the locations where flood defenses were insufficient (i.e., failed to prevent socioeconomic impacts) were identified in the HANZE database (*30*, *31*), containing information with NUTS3 regions that were reported as affected based on available historical sources. Other NUTS3 with potentially significant flooding identified in the modeled flood catalog were considered to be protected from flooding. The exact factual flood protection levels are estimated from the vine-copula models of Paprotny *et al.* (*34*). Factual and counterfactual protection levels, defined as the probability of the hydrological event causing socioeconomic impacts, were modeled both at the event level and for each NUTS3 region constituting the event’s impact zone. The vine-copula models were trained on historical data of flood impact occurrence and nonoccurrence within the modeled flood catalog, using a set of hydrological and socioeconomic predictors (table S3). An event was considered prevented by defenses in the counterfactual scenario if the model correctly identified it as a case of flooding and the mean counterfactual model prediction indicated no flooding. At the more detailed regional level (an event consists of one or more regions, affected or not), counterfactual predictions of impacts were only considered for regions that were correctly classified by the model under factual conditions as “flooding” or “no flooding.” This is a relatively conservative assumption but increases the confidence in the results by not considering cases where the model makes incorrect predictions for the factual scenario.

The uncertainty was sampled from the probabilistic output of the vine-copula models. The change in impacts due to uncertainty of impacts at the NUTS3 level was assessed using potential impact with depth-damage functions (*42*, *79*) without applying the vine-copula vulnerability models (see the “Flood vulnerability” section below). This was done to avoid reapplying the vine-copula model for each sample of possible flood footprints, which would be too computationally demanding.

### Flood vulnerability

For each event, within the historical impact zone, vulnerability was estimated with a two-tier approach. First, depth-damage functions were used to derive a static prediction of fatalities (*79*) and economic losses for six asset classes (*42*). Then, a set of four vine-copula models presented in Paprotny *et al.* (*34*) was applied to correct the estimate of fatalities, population affected, and economic loss to account for local socioeconomic conditions. The vine-copula models were built using data on historical, reported impacts in HANZE (*30*) converted to relative impacts using potential modeled impact (*31*). A set of hydrological and socioeconomic predictors was used to predict the changes in flood vulnerability in space and time and then applied to all events in this study (see table S3 for a list of predictors). Where available, reported impacts were used, while any gaps in the data were filled with the model predictions. Two of the four vine-copula models were derived to estimate fatalities, one to derive the probability of any fatalities (as a large share of events had no fatalities), and the other to derive the magnitude of fatalities. However, in most cases (all 1504 events from the HANZE database), the occurrence or nonoccurrence of fatalities was known; hence, only the second model was applied to estimate the magnitude of impacts. Otherwise, for the remaining 225 events, the estimated fatalities integrate the probability of fatality occurrence and estimated magnitude. As one of the parameters in the vulnerability models is the total relative impact of the event, if, under a certain counterfactual scenario (changes in climate, catchment alteration, or flood protection level), additional regions are affected beyond the factual ones, the vulnerability predicted by the vine-copula model under that scenario is used only for those additional regions, and not for the factual impact zone, to maintain a consistent estimate of vulnerability.

The vulnerability models from Paprotny *et al.* (*34*) were reapplied under 1950 socioeconomic conditions and flood experience (*30*, *31*, *33*). For floods with zero reported fatalities, they were possible under counterfactual conditions, if the mean counterfactual prediction of the vine-copula model was “fatalities,” but the factual prediction was correctly identified as “no fatality.” For floods with reported fatalities, the reverse approach was applied: Only floods with correct factual model predictions were allowed to transition to a no fatality counterfactual. As for flood protection, this is a relatively conservative assumption but increases the confidence in the results. The uncertainty was sampled from the probabilistic output of the vine-copula models.
