# Supplementary material for: Attribution of flood impacts shows strong benefits of adaptation in Europe since 1950
Source: Sci Adv. 2025 Aug 15;11(33):eadt7068. doi: 10.1126/sciadv.adt7068 (PMC12356248; doi:10.1126/sciadv.adt7068)
Supplement: Supplementary file 1 — Figs. S1 to S6 Tables S1 to S3 [file sciadv.adt7068_sm.pdf]

Science Advances
AAAS

Supplementary Materials for

Attribution of flood impacts shows strong benefits of adaptation in Europe since 1950

Dominik Paprotny *et al.*

Corresponding author: Dominik Paprotny, dominik.paprotny@pik-potsdam.de

Sci. Adv. **11**, eadt7068 (2025)
DOI: 10.1126/sciadv.adt7068

This PDF file includes:

Figs. S1 to S6
Tables S1 to S3

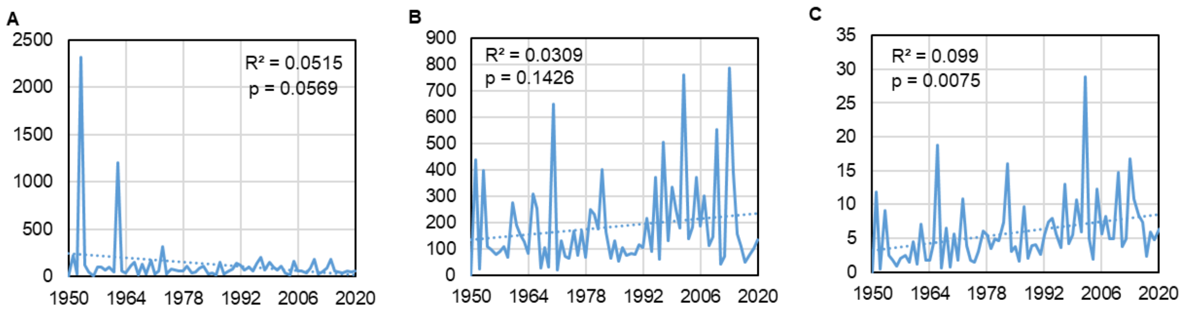

Supplementary Fig. S1. Annual distribution of impacts of European floods. The panels indicate fatalities (A), persons affected in thousands (B) and economic losses in billions of 2020 euros (C). The dotted line denotes the linear trend.

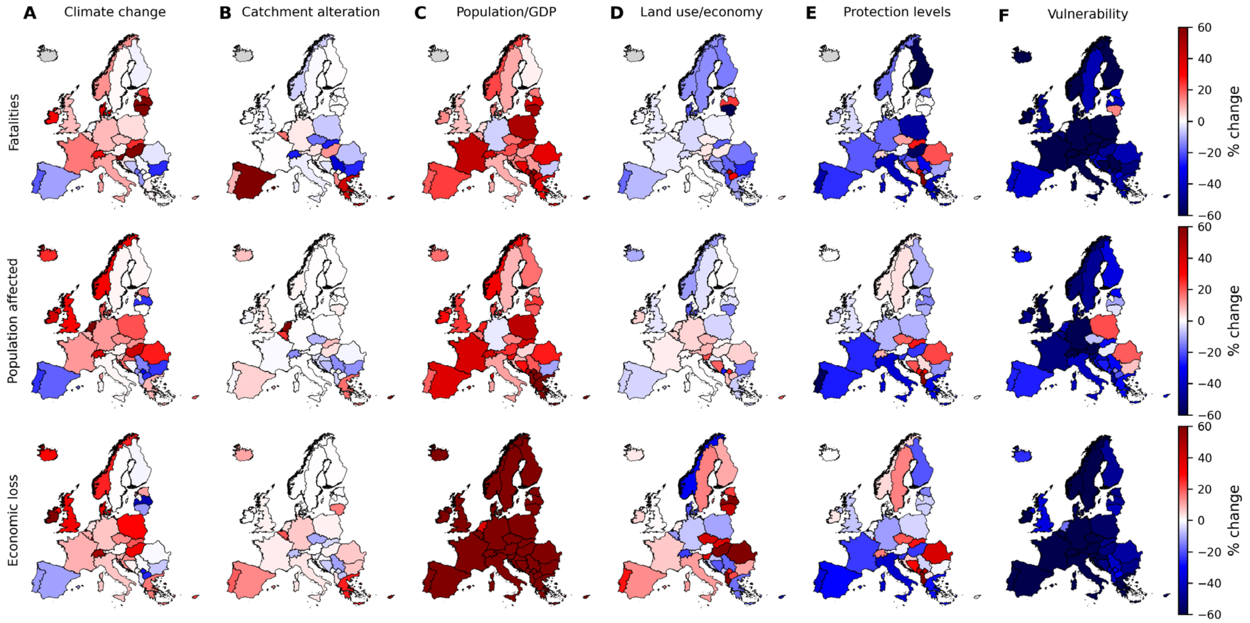

Supplementary Fig. S2. Attribution of impacts by country and driver (% change). Contribution to impacts (in rows) of different drivers (columns **A-F**), by country, expressed as % change of factual impacts relative to the counterfactual scenario of no change in the individual driver since 1950. Each panel represents one driver of change.

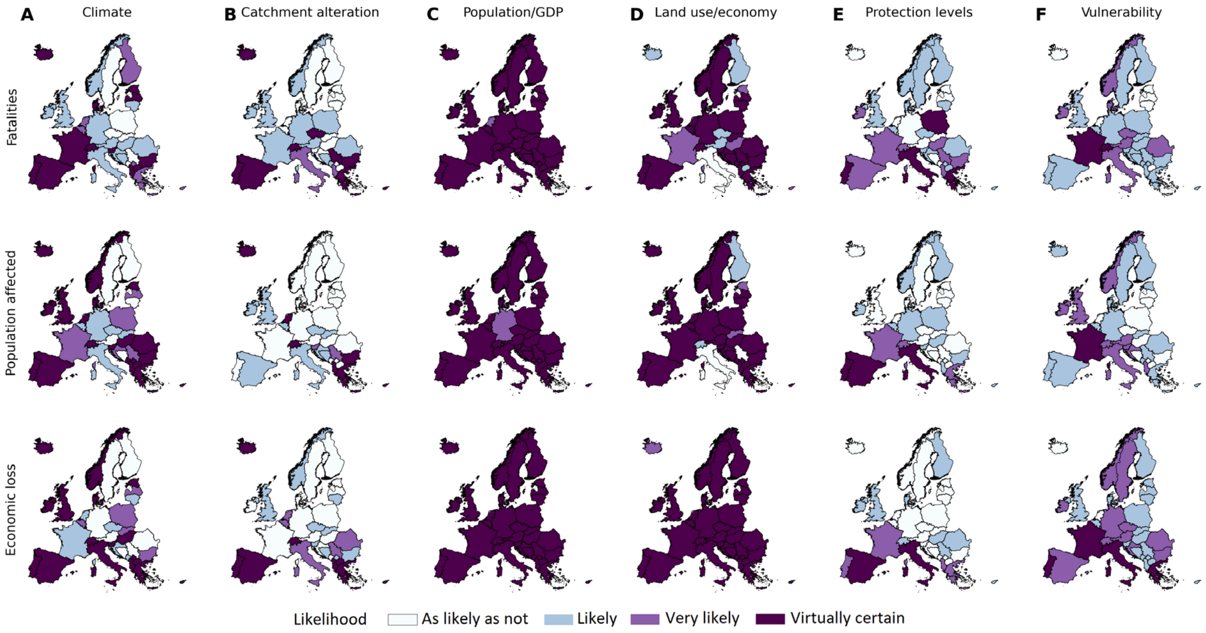

Supplementary Fig. S3. The confidence in the attribution of impacts by country and driver. Contribution to impacts (in rows) of different drivers (columns **A-F**), by country, as likelihood class. The likelihood that the difference between factual and counterfactual scenarios is larger than the model uncertainty (see fig. S2): as likely as not (<66%), likely (66-90%), very likely (90-99%), virtually certain (>99%).

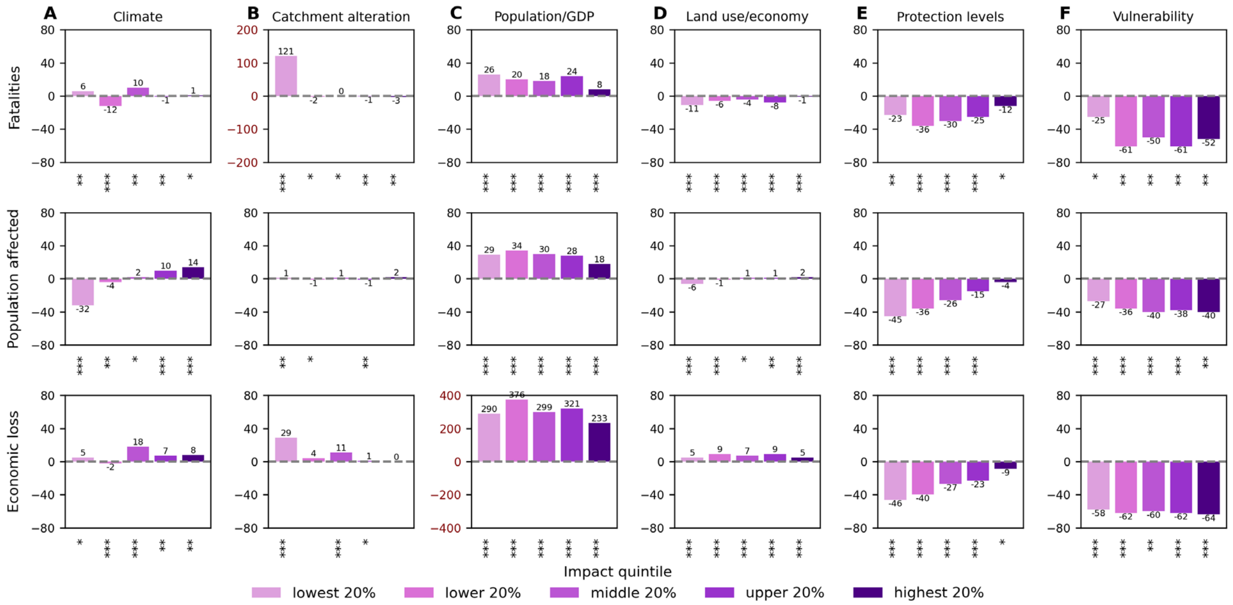

Supplementary Fig. S4. Attribution of impacts by size of flood and driver (% change). Contribution to impacts (in rows) of different drivers (columns **A-F**), by size of flood (quintile of modelled flooded area), expressed as % change of factual impacts relative to the counterfactual scenario of no change in the individual driver since 1950. Asterisks under each bar represent the likelihood that the difference between the factual and counterfactual scenario is larger than the model uncertainty: likely (*), very likely (**) and virtually certain (***).

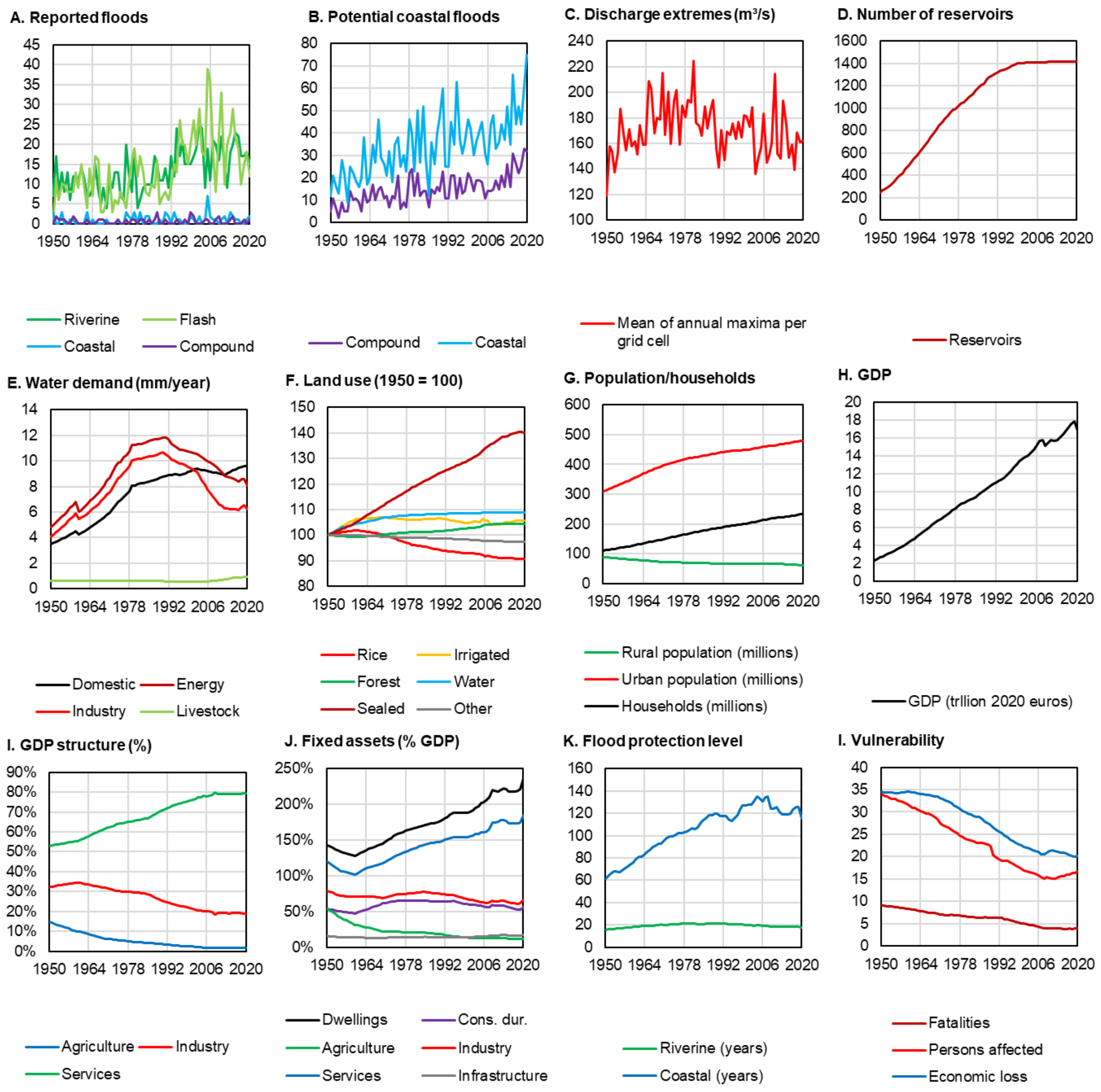

Supplementary Fig. S5. Selected socioeconomic trends across the study domain. (A) number of reported floods by type (*29*); **(B)** modelled number of potential coastal and compound events (*30*); **(C)** Means of annual maxima of river discharges per grid cell (*31*); **(D)** number of existing reservoirs (*31*); **(E)** water demand in mm per year and grid cell (*31*); **(F)** change in land use types relevant for hydrological modelling relative to year 1950 (*31*); **(G)** total population and households, in millions (*32*); **(H)** gross domestic product in trillion euro in 2020 prices and exchange rates (*32)*; **(I)** structure of gross domestic product by sector (*32*); **(J)** fixed assets by sector relative to GDP in % (*32*); **(K)** estimated average flood protection level by type of flood as return period in year (*33*); **(L)** vulnerability (relative loss) of a hypothetical average damaging flood by impact type in % of potential loss (*33*).

.

Drivers	Climate change	Catchment alteration	Population and economic growth	Land use and economic structure	Flood protection levels	Flood vulnerability
Factual impact estimate (conditions at the time of the event)	Extreme river discharges and sea levels reconstructed with climate reanalysis and hydrological models with derivation of 100-m flood inundation zone	River discharge simulation includes the effect of historical changes in land use, reservoir capacity and water demand	Historical population and gross domestic product (GDP) at the level of subnational regions	High-resolution model reconstruction (100 m) of the historical spatial distribution of population and fixed assets by economic sector	Regions with adequate (not impacted by the event) and inadequate (impacted by the event) flood protection levels	Estimated regional vulnerability (relative loss) using multivariate vine-copula models for different impact types
Counterfactual impact estimate (conditions in year 1950)	Detrended river discharge and storm surge heights through transformed-stationary extreme value analysis used to derive 100-m flood inundation zone	River discharge simulation without changes in land use, reservoir capacity and water demand after 1950	Population and GDP at the level of subnational regions in 1950	Model reconstruction of the spatial distribution of population and assets in 1950	Estimated regional flood protection level under hydrological and socioeconomic conditions of 1950 using multivariate vine-copula models	Estimated regional vulnerability under hydrological and socioeconomic conditions of 1950
Uncertainty analysis	Uncertainty of impact estimation due to uncertainty of hydrological models	Uncertainty of impact estimation due to uncertainty of hydrological models	Uncertainty of historical socioeconomic statistical data	Probabilistic output of the land-use model	Probabilistic output of the vine-copula models	Probabilistic output of the vine-copula models

Supplementary Fig. S6. Summary of the methodology. The graph highlights six drivers and the derivation of the factual and counterfactual estimate of each event's impacts.

Supplementary Table S1. Distribution of flood impacts in Europe. Number of flood events in the attribution dataset by type, decade and country (only those with the highest number of events).

Decade	Riverine	Flash	Coastal	Compound	Total
1950	99	57	8	8	172
1961	97	59	12	7	175
1971	95	74	10	5	184
1981	107	82	17	8	214
1991	167	93	10	9	279
2001	241	129	15	5	390
2011	181	111	16	7	315
Total	987	605	88	49	1729
Country	Riverine	Flash	Coastal	Compound	Total
Italy	202	181	5	20	408
Spain	81	114	1	1	197
France	82	71	8	11	172
United Kingdom	63	27	25	4	119
Germany	51	22	4	1	78
Greece	26	38	0	1	65
Switzerland	22	28	0	0	50
Romania	40	6	0	1	47
Serbia	34	10	0	0	44
Czechia	28	14	0	0	42
Poland	34	6	1	1	42
Albania	28	12	0	1	41
Other countries	296	76	44	8	424

Supplementary Table S2. Gap-filling impact data. Reported historical flood events in the HANZE database, detailed whether they were reconstructed in the modeling chain (first column) and whether their impacts were reported in historical sources or were gap-filled with model estimates.

Inclusion in this study	Impacts	Source of impact data	Fatalities		Population affected		Economic loss	
			Events	Persons	Events	Thousand persons	Events	Billion 2020 euros
Included	Reported	HANZE database	1496	8299	773	8809	755	280
	Unreported	Model prediction	233	906	956	4410	974	134
	Total		1729	9205	1729	13219	1729	414
Not included	Reported	HANZE database	531	1917	192	334	167	11
	Unreported	Extrapolation	2	16	341	245	366	9
	Total		533	1933	533	579	533	21
All reported flood events			2262	11138	2262	13798	2262	434
Events/impacts included here as % of total			76.4%	82.6%	76.4%	95.8%	76.4%	95.3%

Supplementary Table S3. Modelling flood protection and vulnerability. Predictors of flood protection levels and flood vulnerability used in the vine-copula models from Paprotny et al. (*33*).

Target variable	Predictor variables
Flood impact (event level)	average return period<br>event duration<br>average annual potential modelled economic loss relative to regional GDP<br>GDP per capita<br>floods in previous 20 years<br>share of GDP in agricultural sector
Flood impact (region level)	average return period<br>GDP per capita<br>floods in previous 30 years<br>event duration in the region
Mortality (chance of fatalities)	average return period<br>share of urban population<br>share of GDP in services sector<br>share of artificial land other than urban fabric
Mortality (magnitude)	potential modelled fatalities affected of the event relative to regional population<br>share of GDP in the agricultural sector<br>floods in previous 20 years<br>average water depth
Relative population affected	potential modelled population affected of the event relative to regional population<br>GDP per capita<br>average annual potential modelled economic loss relative to regional GDP<br>share of GDP in industry sector<br>share of fixed assets in residential buildings
Relative economic loss	potential modelled economic loss of the event relative to regional GDP<br>GDP per capita<br>floods in previous 30 years<br>share of fixed assets in the agricultural sector<br>egalitarian democracy index
